# Correlation Between Local Air Temperature and the COVID-19 Pandemic in Hubei, China

**DOI:** 10.3389/fpubh.2020.604870

**Published:** 2021-01-18

**Authors:** Cheng-yi Hu, Lu-shan Xiao, Hong-bo Zhu, Hong Zhu, Li Liu

**Affiliations:** ^1^Department of Medical Quality Management, Nanfang Hospital, Southern Medical University, Guangzhou, China; ^2^Department of Infectious Diseases, Nanfang Hospital, Southern Medical University, Guangzhou, China; ^3^Department of Oncology, The First Affiliated Hospital, University of South China, Hengyang, China; ^4^Nanfang Hospital, Southern Medical University, Guangzhou, China

**Keywords:** COVID-19, infectious disease, weather-outbreak correlation, climate and health, temperature, daily new confirmed infections

## Abstract

**Objective:** To clarify the correlation between temperature and the COVID-19 pandemic in Hubei.

**Methods:** We collected daily newly confirmed COVID-19 cases and daily temperature for six cities in Hubei Province, assessed their correlations, and established regression models.

**Results:** For temperatures ranging from −3.9 to 16.5°C, daily newly confirmed cases were positively correlated with the maximum temperature ~0–4 days prior or the minimum temperature ~11–14 days prior to the diagnosis in almost all selected cities. An increase in the maximum temperature 4 days prior by 1°C was associated with an increase in the daily newly confirmed cases (~129) in Wuhan. The influence of temperature on the daily newly confirmed cases in Wuhan was much more significant than in other cities.

**Conclusion:** Government departments in areas where temperatures range between −3.9 and 16.5°C and rise gradually must take more active measures to address the COVID-19 pandemic.

## Introduction

Coronavirus disease 2019 (COVID-19), a severe acute respiratory syndrome caused by coronavirus-2 (SARS-CoV-2), broke out in the city of Wuhan, China, in the early winter of 2019. Since then, it has had a substantial effect on global health, economics, and lifestyles, prompting world governments to take various measures to reduce the damages caused by the outbreak. The pandemic has attracted worldwide attention (1) and many recent studies have focused on the relationships between temperature and COVID-19 (2–4). If such relationships could be determined, corresponding measures could be taken to reduce viral morbidity.

The SARS-CoV-2 is primarily transmitted by fomites or respiratory droplets (5). Environmental factors, such as temperature, have an impact on the survival and spread of viruses transmitted through the respiratory tract (6–8). Therefore, temperature are assumed to have an impact on the spread of COVID-19. In the past, severe acute respiratory syndrome (SARS), the infectious disease caused by another respiratory-borne coronavirus, broke out in November of 2002 in China and spread rapidly throughout Southeast Asia. In previous studies on SARS, a negative correlation was found between local air temperatures and daily new cases of SARS (9). This supports our conjecture that temperature may affect the spread of COVID-19.

Until now, the relationship between temperature and the spread of the COVID-19 has not been clarified. Limited studies have shown that temperature have impacts on the spread of the COVID-19 (10–12). Tosepu et al. (13) found a positive correlation between temperature and the COVID-19 pandemic. Conversely, Prata et al. (14) found that daily cumulative confirmed cases were negatively correlated with temperature. The results of most of the previous studies are not entirely consistent, and the relationships between temperature and the spread of COVID-19 remain controversial (15). Our study is mainly focused on analyzing the impacts of temperature on the spread of COVID-19, with the aim of guiding the prevention and management of COVID-19 transmission in the real world based on empirical data.

Hubei Province is the region in China that has been the most severely affected by COVID-19. It is, therefore, extremely valuable to study the impact of temperature on the spread of the virus in Hubei. In this study, six cities in Hubei Province were selected due to the greater severity of the COVID-19 outbreak in these cities. We collected the daily new confirmed cases (DNCC) and daily local temperature (maximum and minimum) data to analyse the relationship between temperature and the spread of COVID-19. Considering the incubation period from the date of a patient's infection to the onset of symptoms and the time from the onset of symptoms to a clear diagnosis, the date of diagnosis must necessarily follow the date of infection (16). Therefore, the impact of temperature on the spread of the virus can only be manifested after a period of time (including the incubation period and the time from the onset of symptoms to a clear diagnosis). As the incubation period of COVID-19 is typically 1–14 days, the impact of temperature on DNCC would inevitably display a certain lag between infection and diagnosis (17). To clarify the influence of temperature on the COVID-19 pandemic, we analyzed the correlations between the DNCC and the temperature 0–14 days before diagnosis and established regression models to understand trends in these relationships.

## Materials and Methods

The study area included six cities across Hubei Province in China, namely Wuhan, Xiaogan, Huanggang, Suizhou, Jingzhou, and Huangshi. Among all cities in this province, the numbers of patients infected with COVID-19 were the highest in these selected cities. The DNCC were collected between 25 January and 11 February of 2020 from the government websites of each city (Wuhan: http://www.wuhan.gov.cn/; Xiaogan: http://www.xiaogan.gov.cn/; Huanggang: http://www.hg.gov.cn/; Suizhou: http://www.suizhou.gov.cn/; Jingzhou: http://www.jingzhou.gov.cn/; Huangshi: http://www.huangshi.gov.cn/). Temperature factors included two indicators: daily maximum and minimum temperatures (°C). Considering that COVID-19 has an incubation period of 1–14 days, we collected the daily temperature from 11 January to 11 February of 2020. The temperatures of each city were obtained from local weather stations.

In order to clarify any correlations that might exist between temperature and the COVID-19 pandemic in Hubei, we analyzed the correlations between the DNCC and the daily maximum and minimum temperatures from 0 to 14 days before a diagnosis was confirmed. Statistical analyses were performed using SPSS software v. 22.0 (IBM Corp., USA). First, considering the small sample size, we conducted a Shapiro–Wilk normality test to analyse the daily maximum and daily minimum temperature data, as well as the DNCC for the six studied cities (Supplementary Table 1). We found that the daily maximum and daily minimum temperature data for these cities were normally distributed, as were the DNCC data from Wuhan, Huanggang, Jingzhou, and Huangshi. We, then, employed Pearson correlation analyses to determine the correlations between the DNCC and the daily maximum temperature (Table 1) and daily minimum temperature (Table 2) from 0 to 14 days prior to the a confirmed diagnosis of COVID-19. As the DNCC data from Xiaogan and Suizhou were not normally distributed, we instead used Spearman rank correlations to analyse these data. Through correlation analyses, we were able to identify the days prior to the diagnosis wherein local temperatures were the most strongly correlated with DNCC.

**Table 1:** The correlations between the DNCC and daily maximum temperature 0–14 days prior to COVID-19 diagnoses in Hubei.

**Day**	**Wuhan**		**Xiaogan**		**Huanggang**		**Suizhou**		**Jingzhou**		**Huangshi**	
	**CC**	***P***	**CC**	***P***	**CC**	***P***	**CC**	***P***	**CC**	***P***	**CC**	***P***
0	0.312	0.207	0.548	0.019	0.655	0.003	0.383	0.117	0.313	0.207	0.592	0.010
1	0.370	0.131	0.621	0.006	0.510	0.031	0.711	0.001	0.399	0.101	0.612	0.007
2	0.467	0.051	0.610	0.007	0.396	0.104	0.558	0.016	0.501	0.034	0.665	0.003
3	0.607	0.008	0.543	0.020	0.364	0.137	0.430	0.075	0.582	0.011	0.696	0.001
4	0.761	<0.001	0.473	0.047	0.225	0.370	0.243	0.332	0.538	0.021	0.585	0.011
5	0.668	0.002	0.285	0.251	−0.197	0.434	0.127	0.615	0.217	0.386	0.276	0.268
6	0.531	0.023	0.126	0.620	−0.512	0.030	−0.203	0.420	−0.143	0.571	−0.034	0.895
7	0.443	0.065	−0.022	0.930	−0.610	0.007	−0.387	0.112	−0.276	0.261	−0.238	0.342
8	0.370	0.130	−0.125	0.547	−0.317	0.200	−0.256	0.304	−0.310	0.210	−0.162	0.520
9	0.289	0.246	−0.073	0.772	0.035	0.889	−0.251	0.316	−0.247	0.323	−0.095	0.706
10	0.175	0.487	0.036	0.887	−0.064	0.800	−0.041	0.871	−0.187	0.458	0.049	0.848
11	0.035	0.889	−0.171	0.497	−0.018	0.942	0.215	0.392	−0.140	0.580	−0.002	0.993
12	−0.063	0.803	−0.229	0.361	0.063	0.805	0.252	0.313	−0.111	0.660	−0.173	0.491
13	−0.073	0.773	−0.115	0.650	0.156	0.536	0.304	0.221	0.077	0.760	0.076	0.763
14	0.026	0.920	0.151	0.548	0.240	0.338	0.352	0.152	0.356	0.147	0.292	0.239

*CC, correlation coefficient*.

**Table 2:** The correlations between the DNCC and daily minimum temperature 0–14 days prior to COVID-19 diagnoses in Hubei.

**Day**	**Wuhan**		**Xiaogan**		**Huanggang**		**Suizhou**		**Jingzhou**		**Huangshi**	
	**CC**	***P***	**CC**	***P***	**CC**	***P***	**CC**	***P***	**CC**	***P***	**CC**	***P***
0	0.168	0.504	0.081	0.750	0.129	0.611	−0.028	0.911	0.206	0.413	0.319	0.196
1	0.037	0.883	−0.035	0.892	−0.017	0.945	−0.308	0.214	0.372	0.129	0.190	0.451
2	0.059	0.817	−0.140	0.579	−0.282	0.256	−0.438	0.069	0.106	0.676	−0.070	0.782
3	−0.165	0.513	−0.159	0.529	−0.625	0.006	−0.209	0.405	−0.157	0.534	−0.445	0.064
4	−0.348	0.157	−0.255	0.307	−0.674	0.002	−0.353	0.151	−0.345	0.161	−0.649	0.004
5	−0.235	0.347	−0.169	0.502	−0.466	0.051	−0.451	0.060	−0.536	0.022	−0.475	0.047
6	−0.344	0.162	−0.263	0.291	−0.264	0.289	−0.141	0.578	−0.423	0.080	−0.320	0.196
7	−0.112	0.659	−0.234	0.351	0.149	0.555	0.107	0.674	−0.213	0.396	−0.150	0.552
8	−0.066	0.795	−0.043	0.866	0.352	0.152	0.260	0.297	−0.016	0.950	−0.063	0.803
9	0.016	0.950	0.148	0.557	0.317	0.200	0.538	0.021	0.154	0.541	0.190	0.450
10	0.168	0.506	0.291	0.241	0.574	0.013	0.499	0.035	0.442	0.066	0.425	0.079
11	0.354	0.149	0.454	0.059	0.650	0.004	0.382	0.118	0.584	0.011	0.617	0.006
12	0.492	0.038	0.524	0.026	0.486	0.041	0.228	0.364	0.597	0.009	0.653	0.003
13	0.735	0.001	0.695	0.001	0.216	0.390	−0.014	0.956	0.117	0.643	0.608	0.007
14	0.618	0.006	0.582	0.011	0.051	0.841	−0.228	0.363	−0.104	0.682	0.498	0.035

*CC, correlation coefficient*.

After correlation analyses, we determined the temperatures corresponding to the days for which there was a statistical significance (*p* < 0.05) in the correlation coefficient between the DNCC and the temperatures. We used DNCC as the dependent variable and the temperature corresponding to the selected days as the independent variable to perform stepwise multiple linear regressions. Finally, we established a multiple linear regression model to analyse the relationship between temperature and the COVID-19 pandemic in Hubei and used relevant parameters to evaluate the reliability of our model.

## Results

### DNCC and Daily Temperatures in Selected Regions

**Figure 1:**
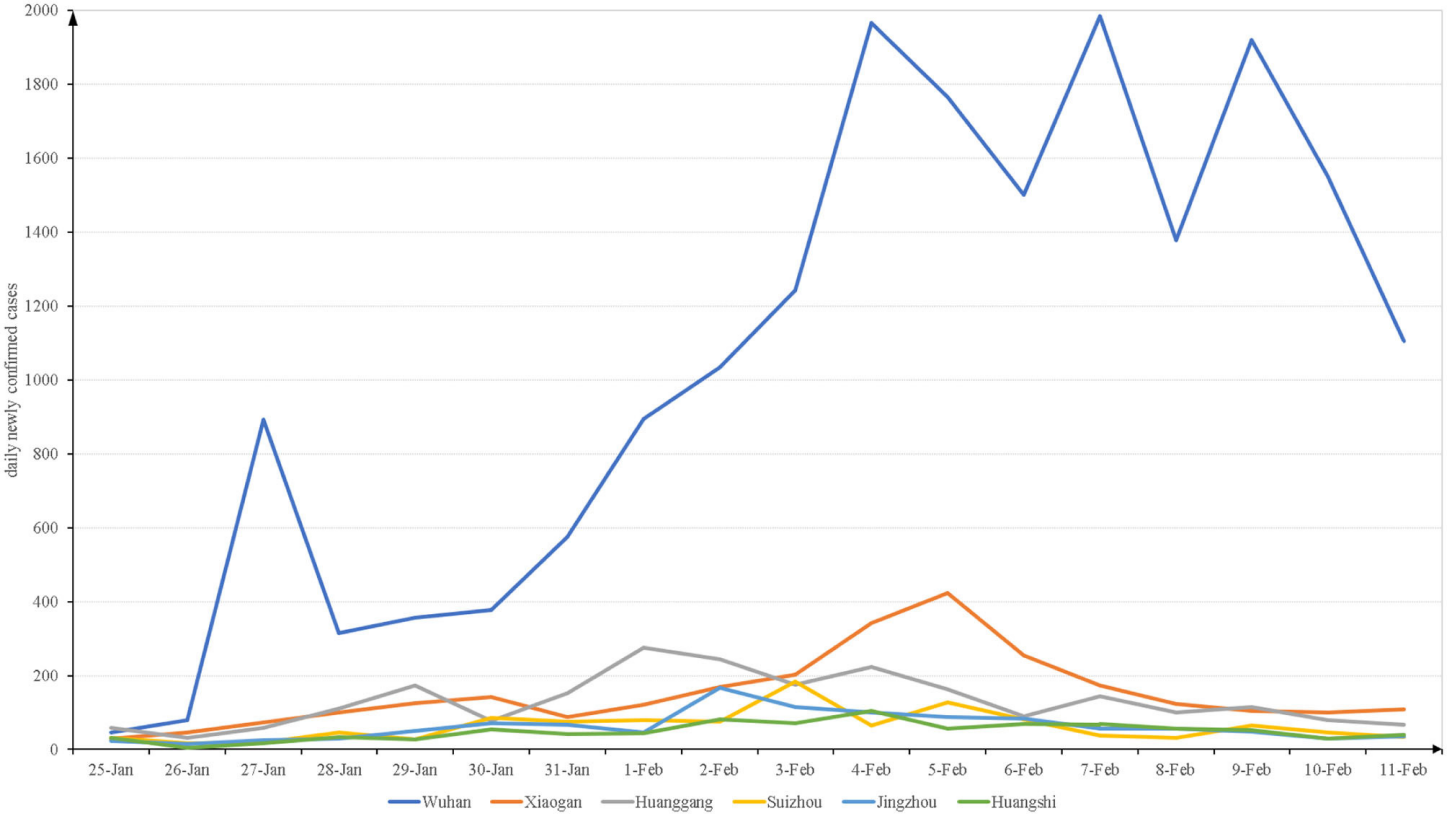
The daily newly confirmed cases from 2020-1-25 to 2020-2-11 in Hubei.

As shown in Figure 1, the trends in the DNCC differed slightly among the six cities investigated. Beginning on 25 January, the DNCC increased in all cities. In Wuhan, Xiaogan, Huanggang, Suizhou, Jingzhou, and Huangshi, the DNCC peaked at *n* = 1,985, 424, 276, 183, 166, and 104 on the 7th, 5th, 1st, 3rd, 2nd, and 4th of February, respectively. After reaching these peaks, the overall trends in the DNCC declined across all cities, although there were some fluctuations.

**Figure 2:**
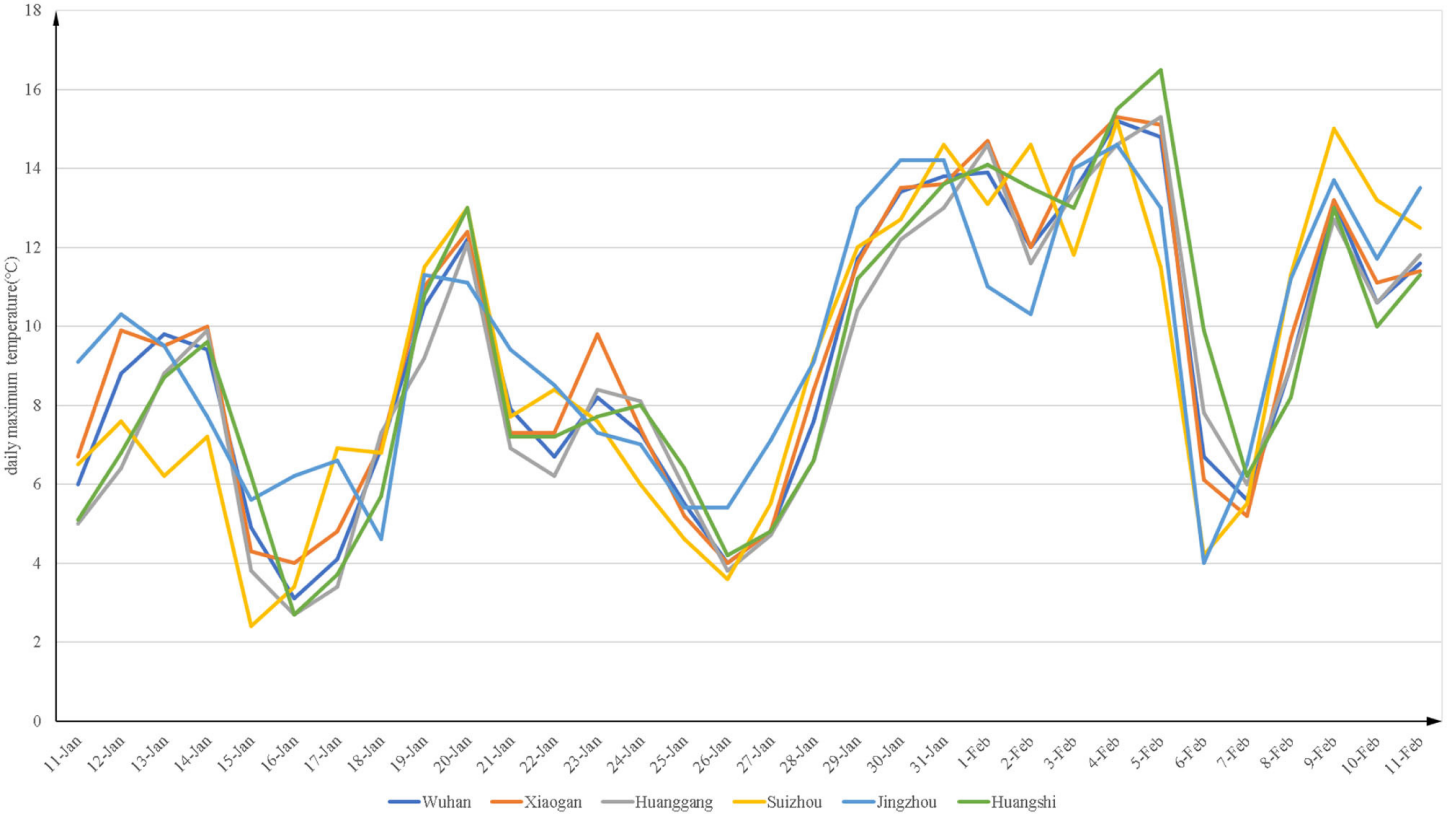
The daily maximum temperature (°C) from 2020-1-11 to 2020-2-11 in Hubei.

**Figure 3:**
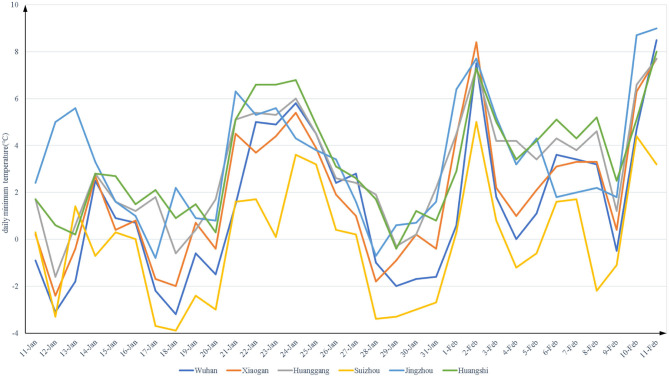
The daily minimum temperature (°C) from 2020-1-11 to 2020-2-11 in Hubei.

Figures 2, 3 show the daily maximum and minimum temperatures from 11 January to 11 February of 2020. The lowest maximum temperature was 2.4°C and the highest was 16.5°C. The lowest minimum temperature was −3.9°C and the highest was 9°C.

### Correlation Between DNCC and Daily Temperature

Tables 1, 2 present the results of Pearson correlation and Spearman rank correlation analyses, depending on the normality of the underlying data. We, first, evaluated the correlations between the DNCC and daily temperature 0–14 days prior to COVID-19 diagnoses in each city and found that the correlations differed among the cities. In Wuhan, the DNCC were positively correlated with the daily maximum and minimum temperatures 3–6 and 12–14 days prior to diagnosis (*p* < 0.05), respectively. In Xiaogan, the DNCC were positively correlated with the daily maximum temperature 0–4 days prior and to the daily minimum temperature 12–14 days prior to diagnosis (*p* < 0.05). In Huanggang, the DNCC were positively correlated with the daily maximum temperature 0–1 days prior and the daily minimum temperature 10–12 days prior to diagnosis (*p* < 0.05), while they were negatively correlated with the daily maximum and minimum temperatures 6–7 and 3–4 days prior to diagnosis (*p* < 0.05), respectively. In Suizhou, the DNCC were positively correlated with the daily maximum temperature 1–2 days preceding diagnosis and to the daily minimum temperature 9–10 days prior (*p* < 0.05). In Jingzhou, the DNCC were positively correlated with the daily maximum and minimum temperatures 2–4 and 11–12 days prior to diagnosis (*p* < 0.05), respectively; meanwhile, they were negatively correlated with the minimum temperature 5 days prior to diagnosis (*p* < 0.05). Finally, in Huangshi, the DNCC were positively correlated with the daily maximum and minimum temperatures 0–4 and 11–14 days preceding diagnosis (*p* < 0.05), respectively, while they were negatively correlated with the daily minimum temperature 4–5 days prior to diagnosis (*p* < 0.05).

### Model Fitting

To further assess the quantitative relationship between DNCC and daily temperatures, stepwise multiple linear regression was used to screen the temperature factors. All temperature data for which *p* < 0.05 in Tables 1, 2 were included in the regression model for further analysis. Table 3 shows the statistical data for the linear regression equation for each city. As shown in Table 3, a one unit increase in the maximum temperature 4 days before the patient was diagnosed positive caused an increase of 129.449 standard deviations of the DNCC in Wuhan. However, an increase of 1°C in the maximum temperature 1 day prior to the diagnosis by was associated with an increase of ~7 in the DNCC in Suizhou. The impact of temperature on the DNCC in Wuhan was much greater than that in other cities. In most cities, an increase in temperature led to an increase in the DNCC, except in Huanggang, where a one unit increase in the minimum temperature 4 days prior to the diagnosis caused a decrease of 16.432 standard deviations in the DNCC.

**Table 3:** Statistical data of the linear regression equation in Hubei.

**Region**	**Model formula**
Wuhan	Y_DNCC_ = −185.716 + 129.449X_max−d4_
Xiaogan	Y_DNCC_ = −24.925 + 12.120 X_max−d0_ + 22.606X_min−d12_ + 17.963X_min−d14_
Huanggang	Y_DNCC_ = 156.597 – 16.432X_min−d4_ + 14.031X_min−d11_
Suizhou	Y_DNCC_ = −7.314 + 6.839X_max−d1_
Jingzhou	Y_DNCC_ = 35.330 + 10.199X_min−d12_
Huangshi	Y_DNCC_ = −21.574 + 3.075X_max−d0_ + 3.820X_max−d3_

*DNCC, daily newly confirmed cases; max-d0, daily maximum temperature on the day when the patient was confirmed; max-d1(3/4), daily maximum temperature 1(3/4) days prior to the diagnosis; min-d4(11/12/14), daily minimum temperature 4(11/12/14) days prior to the diagnosis*.

The linear regression models in our study differed among the cities. In Wuhan, the DNCC were positively correlated with the maximum temperature 4 days preceding the diagnosis, whereas in Xiaogan, the DNCC were positively correlated with the maximum temperature on the day when the patient was confirmed and with the minimum temperature 12 and 14 days prior to the diagnosis. In Huanggang, the DNCC were positively correlated with the minimum temperature 11 days prior to diagnosis and in Suizhou, they were positively correlated with the maximum temperature just 1 day prior to the diagnosis. In Jingzhou, the DNCC were positively correlated with the minimum temperature 12 days prior and in Huangshi, they were positively correlated with the maximum temperature on the day of diagnosis and 3 days prior. Overall, the DNCC in all cities were positively correlated with the maximum temperature ~0–4 days prior to diagnosis or with the minimum temperature ~11–14 days prior.

### Model Evaluation

**Figure 4:**
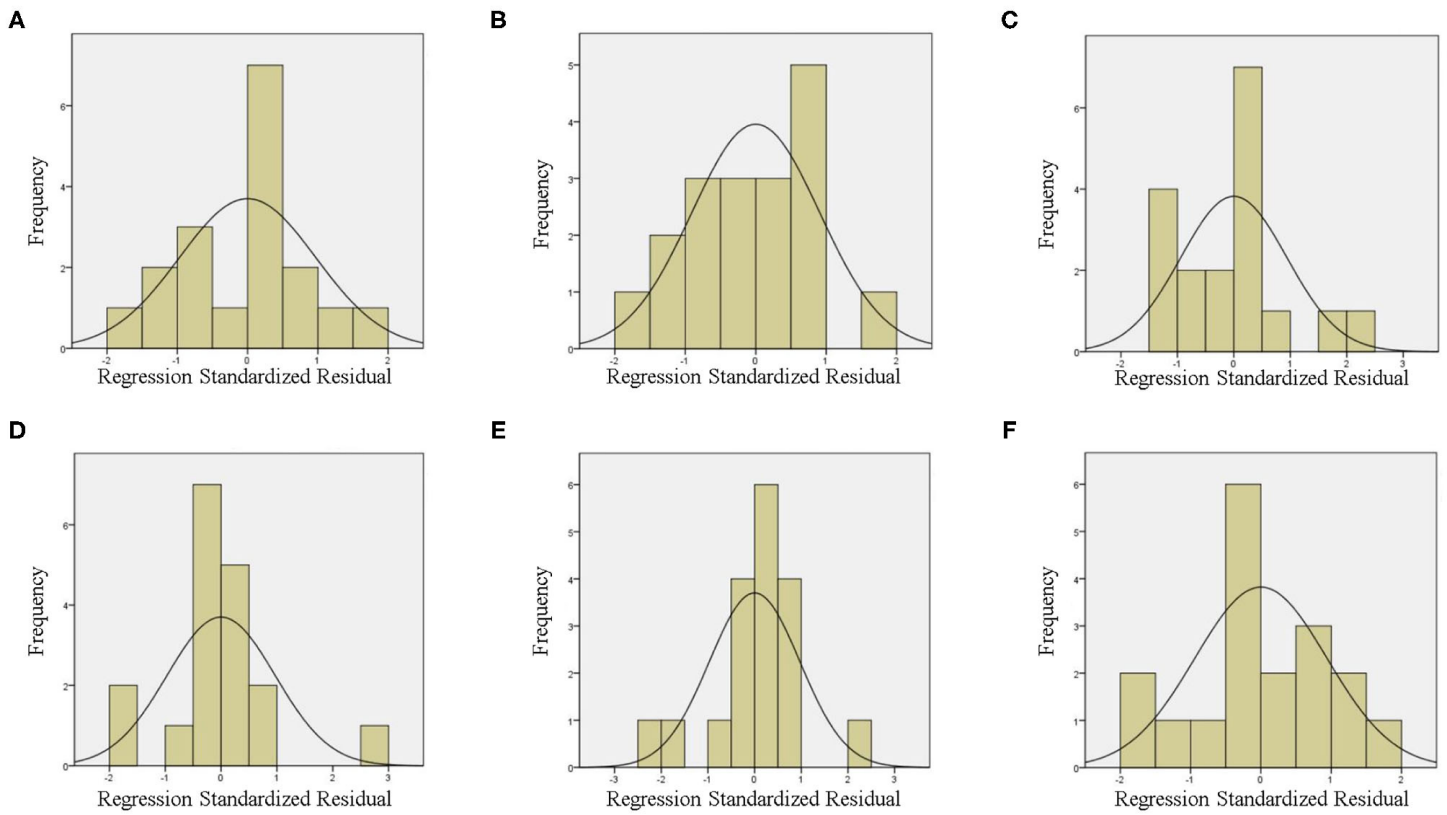
Histogram of the regression standardized residuals of the dependent variable in Wuhan **(A)**, Xiaogan **(B)**, Huanggang **(C)**, Suizhou **(D)**, Jingzhou **(E)**, and Huangshi **(F)**.

**Figure 5:**
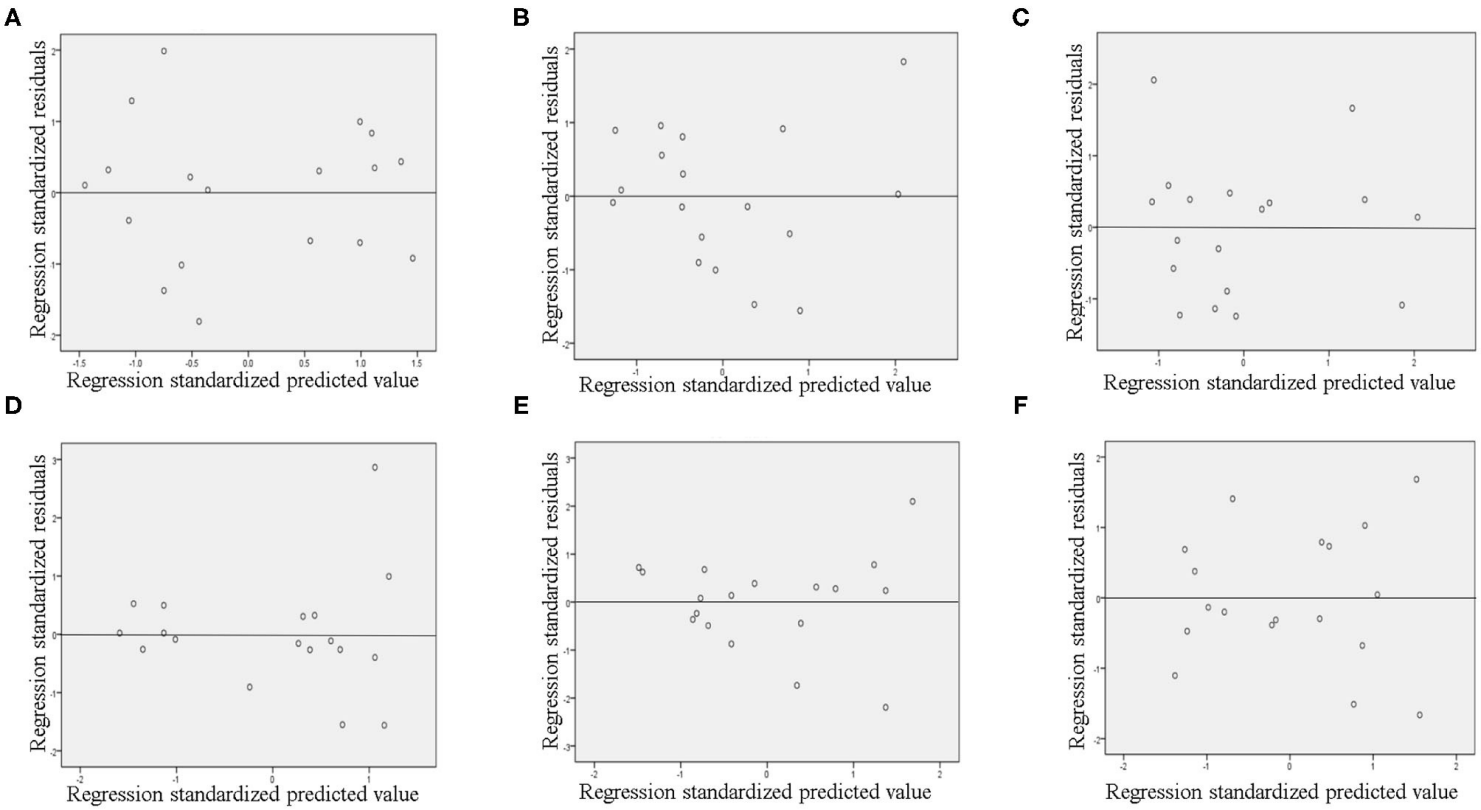
The scatter plot of regression standardized predicted values and regression standardized residuals in Wuhan **(A)**, Xiaogan **(B)**, Huanggang **(C)**, Suizhou **(D)**, Jingzhou **(E)**, and Huangshi **(F)**.

As is well-known, the following four conditions must be met when constructing a linear regression model (18, 19): ① there must be a linear relationship between the independent and dependent variables; ② the residuals must be normally distributed; ③ the residuals must be independent; ④ the residual must exhibit homoscedasticity. In this study, the independent and dependent variables, first, underwent Pearson correlation or Spearman rank correlation analysis, so that they were linearly related. Second, a histogram of the regression-standardized residuals of the dependent variable (Figure 4) showed that the residuals were normally distributed, and the normal P–P plot of the regression-standardized residuals of the dependent variable (Supplementary Figure 1) further demonstrated the normality of the residuals. We, then, found that the residuals were independent by the Durbin–Watson (DW) test because DW ≈ 2 (Table 4), which indicates the absence of autocorrelation (20). Finally, from the scatter plot of regression-standardized predicted values and residuals (Figure 5), we observed that the residuals were randomly distributed and did not increase or decrease as the predicted value increased, indicating that the variance of the residuals was homogeneous and, thus, that our model was reliable.

**Table 4:** The results of model evaluation in Hubei.

	**DW**	**p(F)**	**X**	**p(X)**	**VIF**	***R*^**2**^**	**Adjusted *R*^**2**^**
Wuhan	1.017	<0.001	max-d4	<0.001		0.579	0.553
Xiaogan	1.846	<0.001	max-d0	<0.001	1.007	0.855	0.824
			min-d12	<0.001	1.148		
			min-d14	0.001	1.149		
Huanggang	1.636	0.001	min-d4	0.030	1.346	0.582	0.526
			min-d11	0.049	1.346		
Suizhou	1.754	0.002	max-d1	0.002		0.462	0.429
Jingzhou	0.711	0.009	min-d12	0.009		0.356	0.316
Huangshi	2.719	<0.001	max-d0	0.005	1.042	0.698	0.657
			max-d3	0.001	1.042		

*DW, Durbin-Watson test; F, F test of multiple linear regression model; p(F), the significance of F test; X, independent variable; p(X), the significance of the partial regression coefficient of the corresponding independent variable; VIF, variance inflation factor; R^2^, coefficient of determination*.

To evaluate our model further, we conducted *F*-tests, for which the results were <0.05 for all cities, suggesting that our model was successfully constructed. As shown in Table 4, the minimum adjusted coefficient of determination (*R*^2^) was 0.316 and the maximum value was 0.824. This indicates that 31.6–82.4% of all factors affecting the DNCC were included in the multiple linear regression models. Therefore, our model was appropriate and reliable. Additionally, we tested the significance of the partial regression coefficients of the independent variables in all models and found that they were all statistically significant. Considering that there were more than one independent variable in the models for Xiaogan, Huanggang, and Huangshi, collinearity diagnostics for independent variables in these models were adopted. The models displayed collinearity when the variance inflation factor was >5 (21, 22). After the analysis, we found no collinearity in our models.

### Verifying Our Results in Other Cities

According to the results of our research in Hubei, the DNCC were positively correlated with the maximum temperature ~0–4 days or the minimum temperature ~11–14 days prior to a confirmed diagnosis of COVID-19. In order to determine whether or not this relationship was universal, we included other cities in the study. Among the cities with higher morbidities near Hubei Province, Shaoyang in Hunan Province, and Xinyang in Henan Province were randomly selected for inclusion in our study. Supplementary Figure 2 shows the trends of the DNCC and temperatures in Shaoyang and Xinyang.

Using Shapiro–Wilk normality tests, we found that the daily minimum temperature in Xinyang was not normal, while other data were normally distributed. Therefore, we used Spearman rank correlation analyses to analyse the correlations between the DNCC and the daily minimum temperatures in Xinyang, and Pearson correlation analyses to assess the correlations between the DNCC and daily temperatures (maximum and minimum) in Shaoyang and daily maximum temperatures in Xinyang. Through these analyses, we evaluated the correlations between the DNCC and daily temperature 0–14 days before a diagnosis of COVID-19 was confirmed.

In Shaoyang, the DNCC were positively correlated with the maximum temperatures on the day of the diagnosis and with the minimum temperature 14 days prior to the diagnosis (Supplementary Table 2). In Xinyang, the DNCC were positively correlated with the maximum temperature 3–5 days and minimum temperature 11–12 days preceding diagnosis, respectively. After including these DNCC and temperature data in the multiple linear regression analysis, we observed that the DNCC in Shaoyang and Xinyang were positively correlated with the minimum temperatures 14 and 12 days prior to the diagnosis, respectively (Table 5).

**Table 5:** Statistical data of the multiple linear regression equation in Shaoyang and Xinyang.

**Statistical** **data**	**Shaoyang**	**Xinyang**
Model	Y_DNCC_ = 2.576 + 0.805X_min−d14_	Y_DNCC_ = 8.639 + 3.658X_min−d12_
Durbin-Watson test	1.931	1.506
p(F)	0.031	0.001
X	min-d14	min-d12
p(X)	0.031	0.001
R^2^	0.259	0.518
Adjusted R^2^	0.213	0.488

*DNCC, daily newly confirmed cases; min-d14, minimum temperature 14 days prior to the diagnosis; min-d12, minimum temperature 12 minimum temperature 14 days prior to the diagnosis; p(F), the significance of F test; X, independent variable; p(X), the significance of the partial regression coefficient of the corresponding independent variable*.

The histogram and normal P–P plot (Supplementary Figure 3) of the regression-standardized residuals and Durbin–Watson tests for Shaoyang and Xinyang suggest that the residuals were normal and independent. From the scatter plot of regression-standardized predicted values and residuals, we observed that the variance in the residuals was homogeneous. Finally, the results of p(F), p(X), and adjusted-*R*^2^ showed that our model was reliable. Considering the models for Shaoyang and Xinyang, the conclusions drawn from the original six cities studied appear to be universal. In most cases, the DNCC were positively correlated with the maximum temperature ~0–4 days or the minimum temperature ~11–14 days prior to the diagnosis.

## Discussion and Conclusions

Over the temperature range of −3.9–16.5°C, our results showed that the DNCC were positively correlated with the maximum temperature ~0–4 days or the minimum temperature ~11–14 days prior to the diagnosis in nearly all selected cities, except for Huanggang. However, Prata et al. (14) found that daily cumulative confirmed cases were negatively correlated with temperature between 16.8 and 27.4°C. In addition, Chen et al. (23) pointed out that the transmissibility of COVID-19 could be lower when the local temperature rised. These results suggest that there might be a temperature range that is optimal for the transmission of COVID-19. If temperatures fall below this range, DNCC and temperature would be positively correlated, whereas if temperatures exceed this range, DNCC and temperature would be negatively correlated.

The influence of temperature on the DNCC differed slightly among the studied cities. In our linear regression model, the influence of temperature on the DNCC in Wuhan was much more significant than in other cities. We considered that the following factors were responsible for the differences in our model results for different areas: geo-social diversity and prevention and control measures implemented by the government. According to local government websites, traffic control and city blockade measures were implemented on 26 January, 30 January, 31 January, 25 January, 2 February, and 3 February of 2020 in Wuhan, Xiaogan, Huanggang, Suizhou, Jingzhou, and Huangshi, respectively. Although the times at which local governments adopted traffic control and city blockade measures were similar, their slight differences may have caused differences in the correlations between the temperature and DNCC, causing our model results to differ. Furthermore, in the early stages of the COVID-19 pandemic in China, the diagnosis of patients was limited by the availability of SARS-CoV-2 nucleic acid detection kit, thereby not meeting the scale of the medical need. Considering that patients were diagnosed using nucleic acid detection kits, the differences in the numbers of these kits allocated to different cities would have affected the DNCC, thereby affecting the results of our model. Other environmental factors, such as humidity and wind speed, may be confounding factors in this study. As our analyses were focused on the impacts of temperature on the DNCC, we did not include these variables. According to previous reports, humidity and wind speed may affect the DNCC; however, these results remain controversial. Behnood et al. (24) found that an increase in relative humidity could increase infection rates. However, Meo et al. (25) suggested that an increase in humidity reduced the DNCC in world's top ten hottest countries. Another previous study showed that higher wind speeds 14 days preceding diagnoses resulted in higher DNCC (26); however, Rendana et al. (27) claimed that lower wind speeds could increase the cases of COVID-19. Therefore, the influences of humidity and wind speed on DNCC require further exploration.

Based on the results of model fitting, the adjusted-*R*^2^ in our study ranged from 0.316 to 0.824. Zhu et al. (15) constructed a multiple linear regression model for which adjusted-*R*^2^ = 0.096–0.639, which is much less than the range determined here. Additionally, they did not analyse the residuals nor the collinearity of the independent variables in their model, which may have limited the effectiveness of their model.

This study has several limitations. First, some environmental factors that might affect the DNCC were not included, such as the wind speed and humidity. Second, socioeconomic status, medical resources, and social policies could also affect the spread of COVID-19; hence, these confounding factors should also be included in future studies. Nevertheless, as limited information is currently available on the relationship between environmental conditions and viral transmission, based on our model results, government departments in areas where temperature ranges between −3.9 and 16.5°C and where temperatures are gradually rising should take more active measures to address the COVID-19 pandemic.

## Data Availability Statement

The original contributions presented in the study are included in the article/Supplementary Material, further inquiries can be directed to the corresponding author/s.

## Author Contributions

C-yH and L-sX designed the research study, analyzed the data, and wrote the paper. H-bZ analyzed the data and revised the manuscript. LL and HZ designed the research study and analyzed the data. All authors contributed to the article and approved the submitted version.

## Conflict of Interest

The authors declare that the research was conducted in the absence of any commercial or financial relationships that could be construed as a potential conflict of interest.

## Supplementary Material

Supplementary Figure 1 The normal P-P plot of the regression standardized residuals of the dependent variable in Wuhan **(A)**, Xiaogan **(B)**, Huanggang **(C)**, Suizhou **(D)**, Jingzhou **(E)**, and Huangshi **(F)**.

Supplementary Figure 2 The daily temperature and the DNCC in Shaoyang and Xinyang.

Supplementary Figure 3 Histogram of the regression standardized residuals of the dependent variable in Shaoyang **(A)** and Xinyang **(D)**, the normal P-P plot of the regression standardized residuals of the dependent variable in Shaoyang **(B)** and Xinyang **(E)**, and the scatter plot of regression standardized predicted values and regression standardized residuals in Shaoyang **(C)** and Xinyang **(F)**.

Supplementary Table 1 The normality test of the data in Hubei.

Supplementary Table 2 The correlations between the DNCC and the daily temperature in Shaoyang and Xinyang.
