# Supplementary material for: Correlation Between Local Air Temperature and the COVID-19 Pandemic in Hubei, China
Source: Front Public Health. 2021 Jan 18;8:604870. doi: 10.3389/fpubh.2020.604870 (PMC7848168; doi:10.3389/fpubh.2020.604870)
Supplement: Supplementary Figure 1 — The normal P-P plot of the regression standardized residuals of the dependent variable in Wuhan (A), Xiaogan (B), Huanggang (C), Suizhou (D), Jingzhou (E), and Huangshi (F). [file Data_Sheet_1.ZIP › Supplementary Table 1.docx]

**Supplementary Table 1**. The normality test of the data in Hubei.

| **Shapiro-Wilk normality test** | **DNCC** | **daily maximum temperature** | **daily minimum temperature** |
| --- | --- | --- | --- |
| **Wuhan** | 0.259 | 0.277 | 0.223 |
| **Xiaogan** | 0.008 | 0.339 | 0.095 |
| **Huanggang** | 0.311 | 0.862 | 0.402 |
| **Suizhou** | 0.011 | 0.181 | 0.065 |
| **Jingzhou** | 0.064 | 0.186 | 0.128 |
| **Huangshi** | 0.979 | 0.227 | 0.427 |

DNCC: daily newly confirmed cases.
