# Supplementary material for: Correlation Between Local Air Temperature and the COVID-19 Pandemic in Hubei, China
Source: Front Public Health. 2021 Jan 18;8:604870. doi: 10.3389/fpubh.2020.604870 (PMC7848168; doi:10.3389/fpubh.2020.604870)
Supplement: Supplementary Figure 1 — The normal P-P plot of the regression standardized residuals of the dependent variable in Wuhan (A), Xiaogan (B), Huanggang (C), Suizhou (D), Jingzhou (E), and Huangshi (F). [file Data_Sheet_1.ZIP › Supplementary Table 2.docx]

**Supplementary Table 2**. The correlations between the DNCC and the daily temperature in Shaoyang and Xinyang.

| **Day** | **daily maximum temperature** | | | | **daily minimum temperature** | | | |
| --- | --- | --- | --- | --- | --- | --- | --- | --- |
|  | **Shaoyang** | | **Xinyang** | | **Shaoyang** | | **Xinyang** | |
|  | **CC** | ***P*** | **CC** | ***P*** | **CC** | ***P*** | **CC** | ***P*** |
| 0 | 0.506 | 0.032 | 0.193 | 0.443 | -0.102 | 0.687 | -0.002 | 0.994 |
| 1 | 0.175 | 0.488 | 0.287 | 0.248 | -0.263 | 0.292 | -0.201 | 0.425 |
| 2 | -0.077 | 0.761 | 0.168 | 0.460 | -0.103 | 0.685 | 0.046 | 0.858 |
| 3 | -0.276 | 0.267 | 0.486 | 0.041 | -0.174 | 0.490 | 0.151 | 0.550 |
| 4 | -0.274 | 0.271 | 0.521 | 0.027 | -0.290 | 0.242 | -0.024 | 0.925 |
| 5 | -0.083 | 0.744 | 0.497 | 0.036 | -0.234 | 0.351 | -0.464 | 0.053 |
| 6 | -0.030 | 0.905 | 0.197 | 0.434 | -0.164 | 0.517 | -0.165 | 0.514 |
| 7 | 0.142 | 0.574 | -0.050 | 0.844 | -0.352 | 0.151 | 0.035 | 0.890 |
| 8 | -0.124 | 0.623 | -0.252 | 0.313 | 0.128 | 0.612 | 0.178 | 0.480 |
| 9 | -0.151 | 0.550 | -0.401 | 0.099 | -0.026 | 0.917 | 0.084 | 0.741 |
| 10 | -0.327 | 0.185 | -0.321 | 0.194 | -0.059 | 0.816 | 0.397 | 0.103 |
| 11 | -0.303 | 0.222 | -0.032 | 0.900 | 0.201 | 0.423 | 0.645 | 0.004 |
| 12 | -0.270 | 0.278 | 0.188 | 0.455 | 0.238 | 0.342 | 0.693 | 0.001 |
| 13 | -0.090 | 0.722 | 0.117 | 0.644 | 0.371 | 0.130 | 0.141 | 0.576 |
| 14 | -0.064 | 0.802 | 0.025 | 0.923 | 0.509 | 0.031 | 0.184 | 0.464 |

**CC**: Correlation Coefficient.
