# Supplementary figures and images for: Correlation Between Local Air Temperature and the COVID-19 Pandemic in Hubei, China
Source: Front Public Health. 2021 Jan 18;8:604870. doi: 10.3389/fpubh.2020.604870 (PMC7848168; doi:10.3389/fpubh.2020.604870)

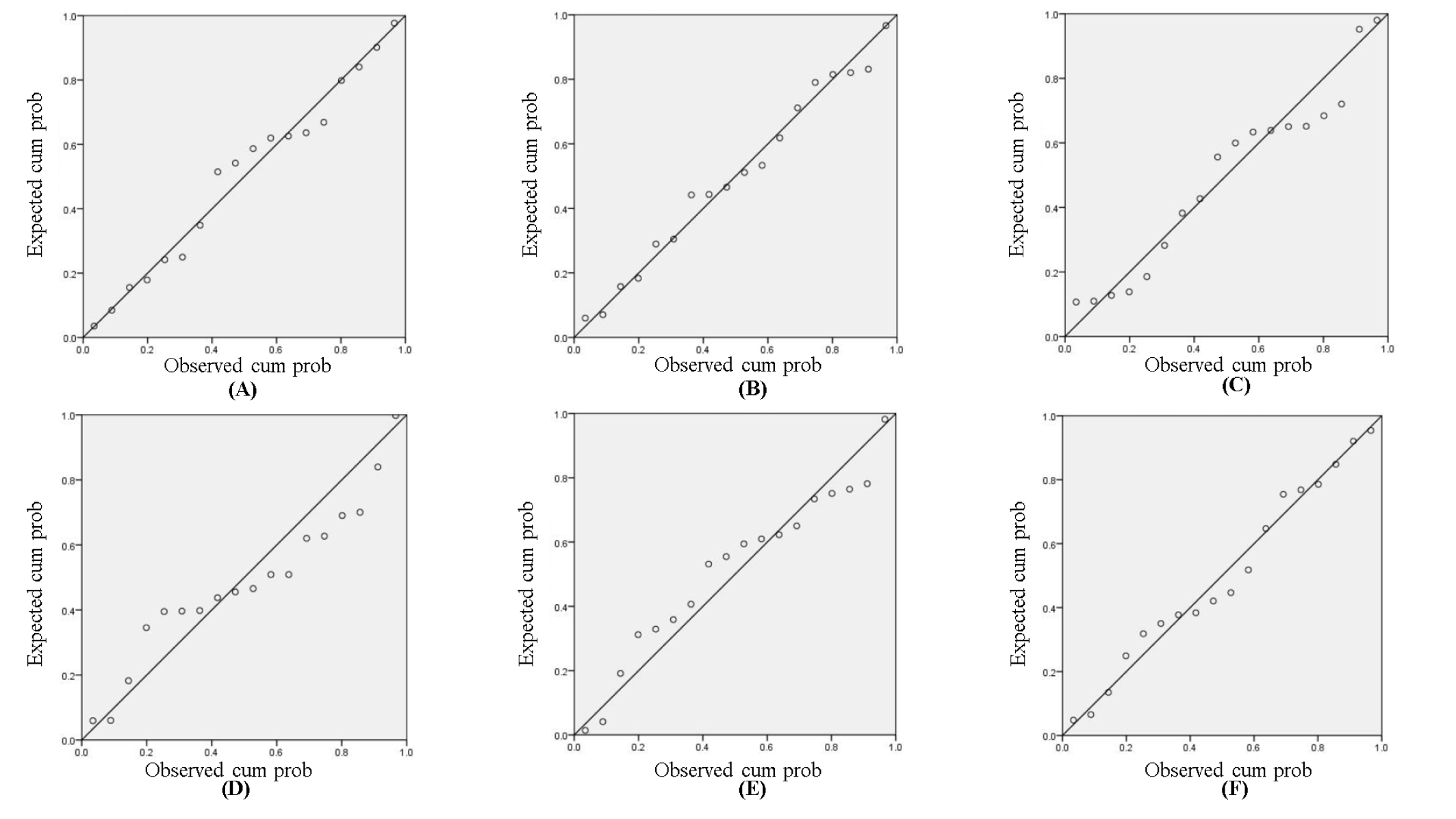

Supplement: Supplementary Figure 1 — The normal P-P plot of the regression standardized residuals of the dependent variable in Wuhan (A), Xiaogan (B), Huanggang (C), Suizhou (D), Jingzhou (E), and Huangshi (F). [file Data_Sheet_1.ZIP › Supplementary Figure 1.tif]

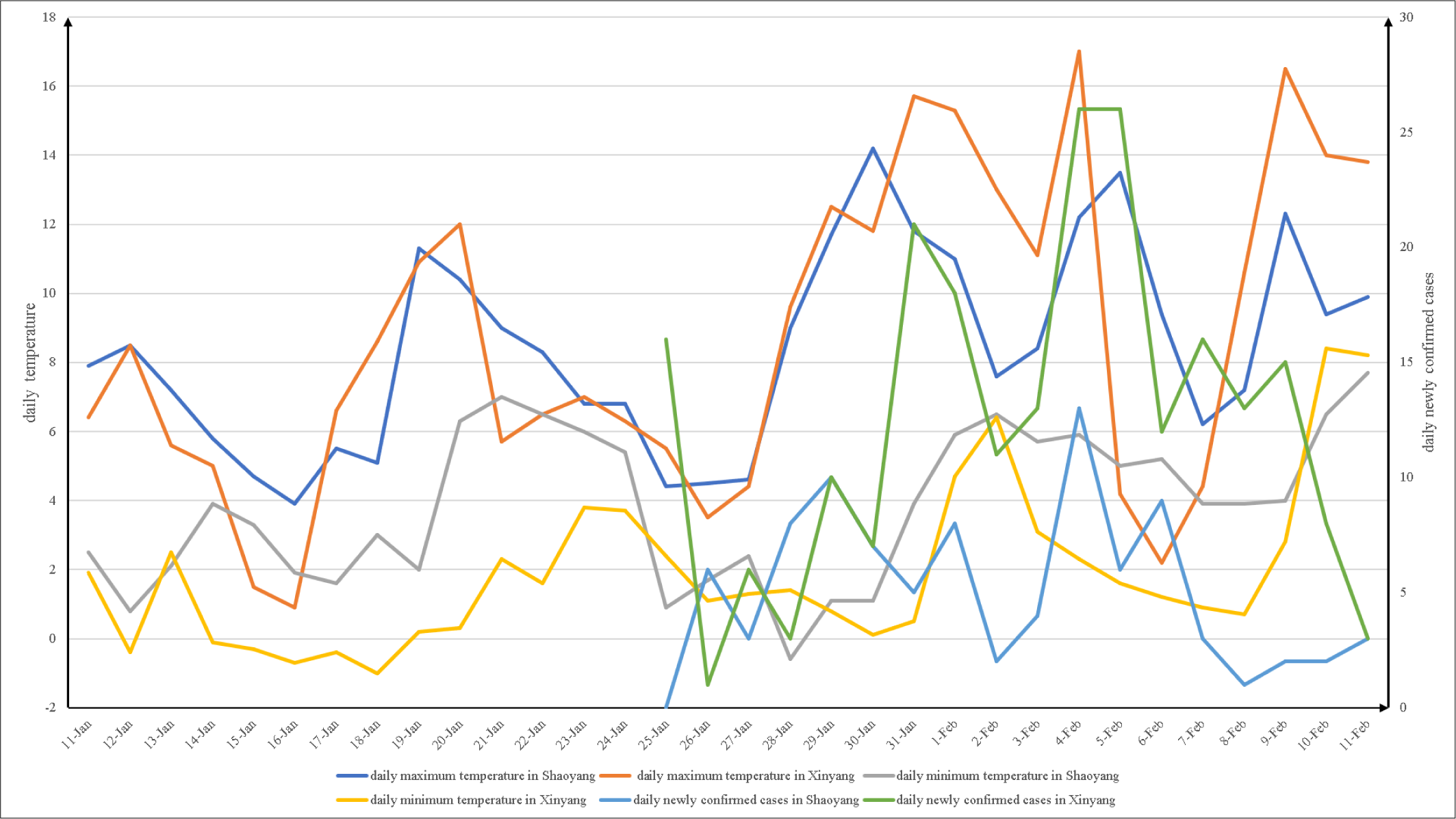

Supplement: Supplementary Figure 1 — The normal P-P plot of the regression standardized residuals of the dependent variable in Wuhan (A), Xiaogan (B), Huanggang (C), Suizhou (D), Jingzhou (E), and Huangshi (F). [file Data_Sheet_1.ZIP › Supplementary Figure 2.tif]

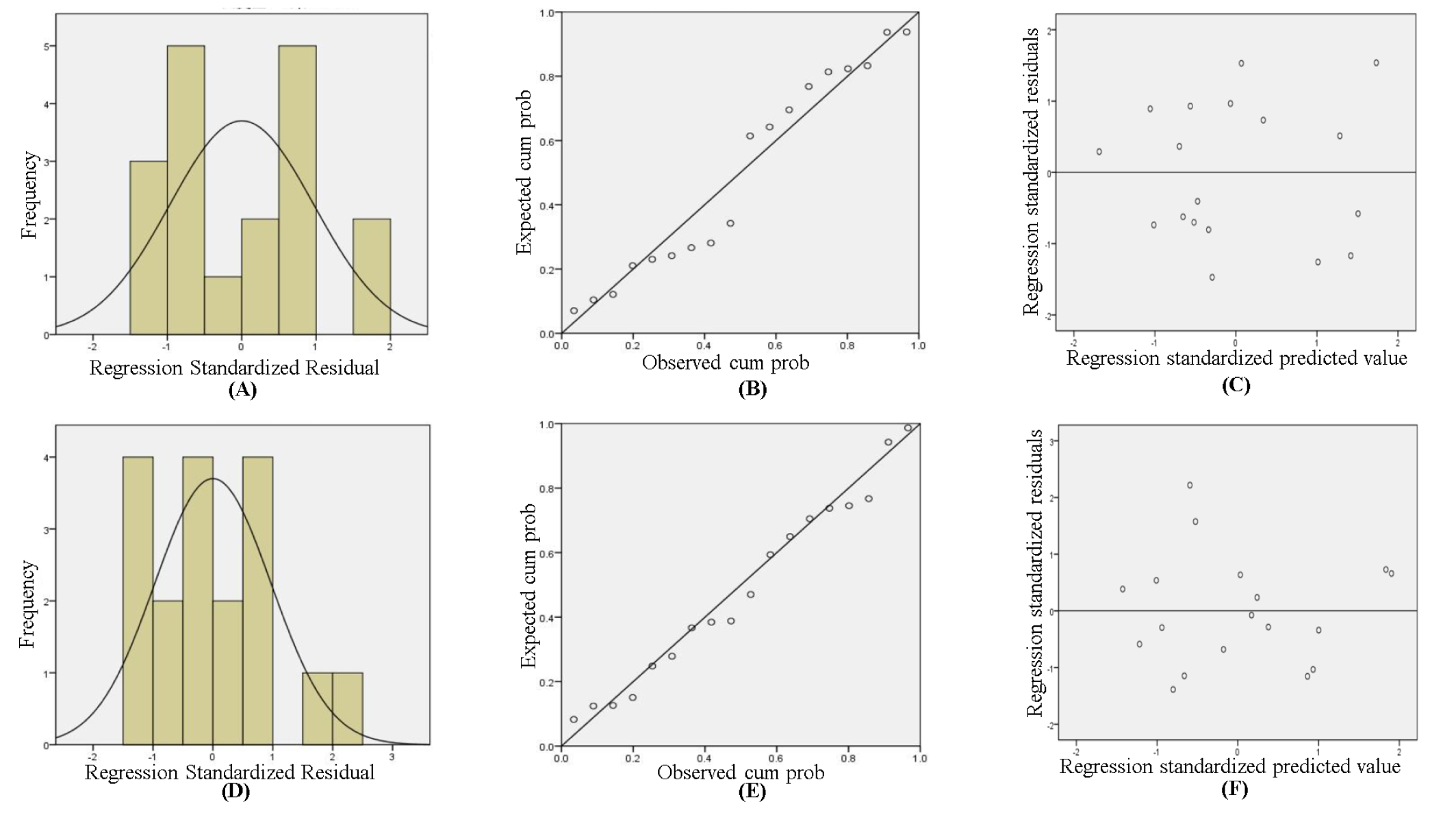

Supplement: Supplementary Figure 1 — The normal P-P plot of the regression standardized residuals of the dependent variable in Wuhan (A), Xiaogan (B), Huanggang (C), Suizhou (D), Jingzhou (E), and Huangshi (F). [file Data_Sheet_1.ZIP › Supplementary Figure 3.tif]
